# Florid Cemento‐Osseous Dysplasia and Simple Bone Cyst Association: Co‐Occurrence or Evolution of the Disease? Two Case Reports and Literature Review

**DOI:** 10.1002/cre2.70459

**Published:** 2026-10-12

**Authors:** Victor Voisin, Paul Galvez, Jean‐Christophe Fricain, Mathilde Fénelon

**Affiliations:** ^1^ Oral Surgery Department CHU Bordeaux Bordeaux France; ^2^ Unité de Formation et de Recherche des Sciences Odontologiques Université de Bordeaux Bordeaux France; ^3^ INSERM, BIOTIS, U1026 Université de Bordeaux Bordeaux France; ^4^ Centre de Compétence des Maladies Rares, Orales et Dentaires Pôle d'Odontologie et Santé Buccale, Centre Hospitalier Universitaire Bordeaux Bordeaux France

**Keywords:** cemento‐osseous dysplasia, florid cemento‐osseous dysplasia, oral surgery, simple bone cyst, solitary bone cyst

## Abstract

**Objectives:**

Cemento‐osseous dysplasia (COD) is the most common benign fibro‐osseous lesion of the jaws, typically asymptomatic and identified through characteristic radiographic features. However, its rare association with simple bone cysts (SBCs) can significantly alter its presentation, mimicking more aggressive jaw lesions. This study aims to report additional cases of COD‐associated SBCs and to review the literature to evaluate whether such lesions may represent a specific manifestation of the COD spectrum.

**Materials and Methods:**

Two cases of florid COD with atypical radiolucencies were described, both exhibiting cortical thinning and focal perforation, as well as bone expansion.

**Results:**

Surgical exploration was performed to rule out a more serious aggressive odontogenic pathology and revealed empty bone cavities, consistent with SBCs.

**Conclusions:**

These cases support the concept of secondary SBCs as part of the COD spectrum. A review of the literature highlights differences in age, sex, location, clinical and radiological features, as well as healing patterns compared to solitary SBCs.

## 1 Introduction

Cemento‐osseous dysplasia (COD) is defined as the most common benign fibro‐osseous lesion of the tooth‐bearing regions of the jaws. It is characterized by a progressive replacement of bone with fibrous tissue containing deposits of cement‐like or osseous material of variable density. It occurs more frequently in middle‐aged women, particularly in certain ethnic groups (Pereira et al. 2016). Four subtypes were defined by the World Health Organization: periapical, focal, florid and familial florid COD (Vered and Wright 2022). Unlike sporadic cases, the familial form is rare and predominantly occurs in younger people, being characterized by more expansile lesions (Toffanin et al. 2000). These subtypes have similar radiographic and histologic appearances, evolving through three distinct stages that correlate with the progressive mineralization of the lesion. The initial, or osteolytic stage, is marked by radiolucent areas located around the apices of teeth. In the intermediate, or mixed/cementoblastic stage, the lesion presents as a mixed radiolucent–radiopaque area due to the appearance of central calcifications. The final, or osteosclerotic stage, is characterized by a dense, well‐circumscribed radiopaque mass surrounded by a thin radiolucent border (Daviet‐Noual et al. 2017; Fenerty et al. 2017).

These lesions are typically asymptomatic and associated with vital adjacent teeth and intact overlying gingiva (Waldron 1993). As a result, they are often discovered incidentally during routine radiographic evaluations (Massereau et al. 2015). Diagnosis is thus usually based on their characteristic clinical and radiographic features. In the absence of symptoms, neither biopsy nor treatment is necessary, and regular dental follow‐up is recommended.

Although rarely reported in the literature, the co‐occurrence of COD and simple bone cysts (SBCs) may significantly alter the classic radiographic features of these otherwise well‐characterized fibro‐osseous lesions, potentially complicating diagnosis. An SBC is a rare, non‐odontogenic intraosseous empty cavity devoid of epithelial lining and often fluid‐filled (Jiang et al. 2021). The first report of an association between COD and SBC was published by Melrose et al. (1976). They noted that SBCs in patients with COD appeared to differ from the more common forms of solitary SBCs, frequently exhibiting active expansion and an unpredictable response to standard treatment. Since that initial observation, only a few similar cases have been documented. Some authors have further proposed that SBCs occurring in the context of COD may represent a distinct clinical and radiological entity, differing from isolated SBCs in both presentation and treatment outcomes (Hajjami et al. 2023). Finally, although a few hypotheses have been proposed to explain this association, its underlying pathogenesis remains unclear.

The aim of this study is twofold. First, we report two new cases of COD‐associated SBCs, highlighting their highly unusual radiographic features, which may mimic other jaw lesions. Second, based on the available literature, we discuss the differences in biological behavior between solitary and COD‐associated SBCs, to explore whether SBCs should be considered a potential manifestation of the COD spectrum, rather than a distinct pathological entity.

## 2 Report of Cases

### 2.1 Case 1

A 43‐year‐old woman of Martinican origin, with no significant medical history, was referred to the Department of Oral Surgery at Bordeaux University Hospital by her general dentist following the incidental discovery of multiple asymptomatic radiolucent mandibular lesions. Clinical examination revealed a firm vestibular swelling adjacent to tooth #46, with intact overlying mucosa. Axial percussion testing of the mandibular teeth was negative. All teeth responded positively to pulp vitality testing, except tooth #32, which had undergone prior endodontic treatment.

Initial imaging, including panoramic radiography and cone‐beam computed tomography (CBCT), revealed multiple mandibular lesions (Figure 1). Mixed radiolucent–radiopaque lesions were observed around the apices of the left mandibular molars, consistent with florid cemento‐osseous dysplasia (FCOD). In contrast, two well‐defined, homogeneous radiolucent lesions were identified in the right posterior mandible. The first was a large multilocular radiolucency measuring approximately 5 cm, extending from tooth #32 to #46, with internal septations and irregular margins. The second was a smaller, well‐circumscribed unilocular lesion near tooth #48. Both lesions showed thinning and focal perforation of the buccal cortical plate, suggesting a more aggressive radiographic appearance. No signs of root resorption were detected.

**Figure 1:**
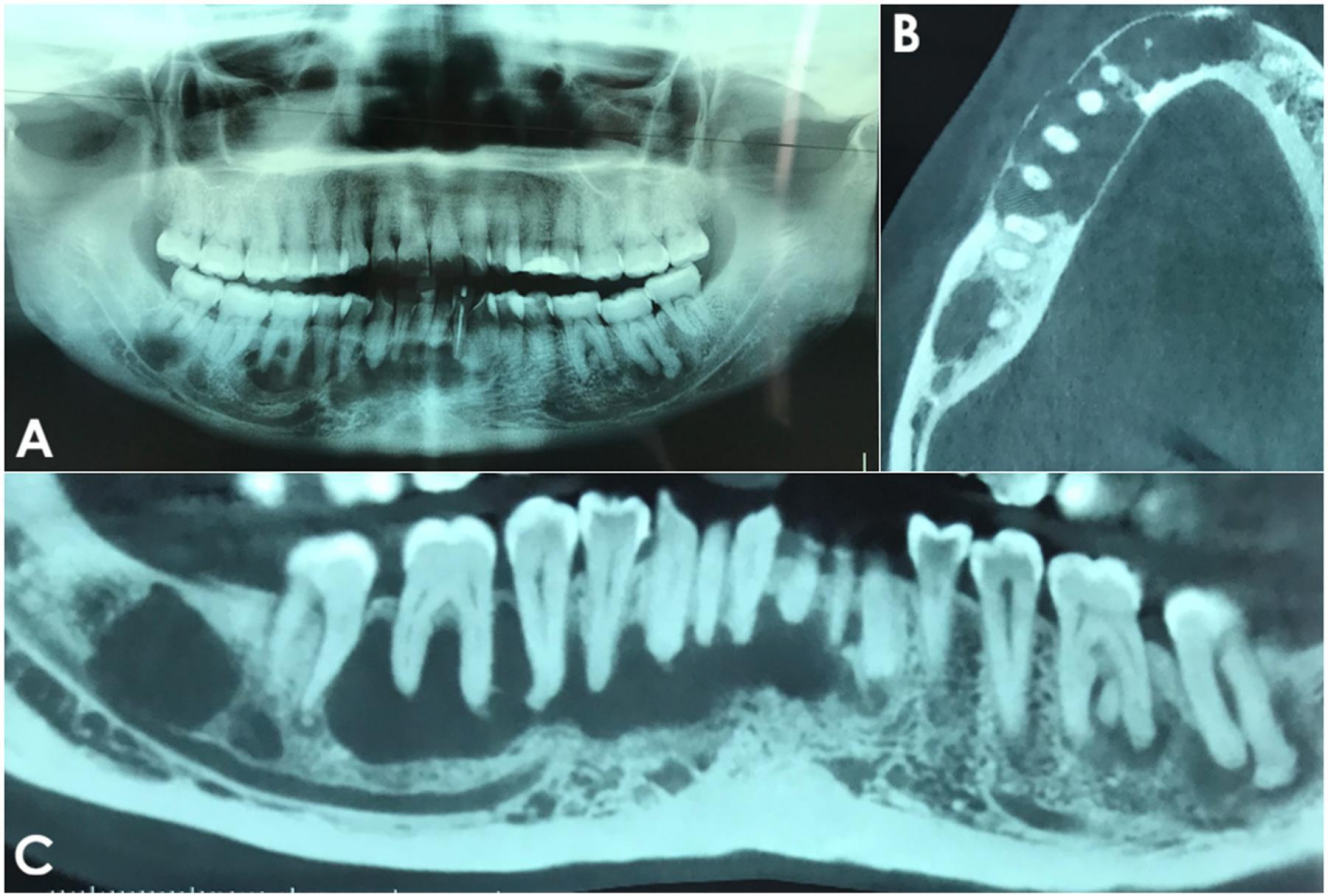
Case 1. Panoramic radiograph (A), axial slice (B), and panoramic reconstruction (C) of the cone‐beam CT.

While the left‐sided lesions exhibited characteristic features of asymptomatic FCOD, the right‐sided radiolucencies raised differential diagnostic concerns, including ameloblastoma or keratocyst, due to their atypical and more aggressive presentation. Surgical exploration of the right mandibular lesion was therefore undertaken under local anesthesia (Figure 2). The overlying buccal cortical bone displayed a bluish hue, and upon removal of a bone window, an empty intraosseous cavity without epithelial lining was found, consistent with an SBC associated with FCOD.

**Figure 2:**
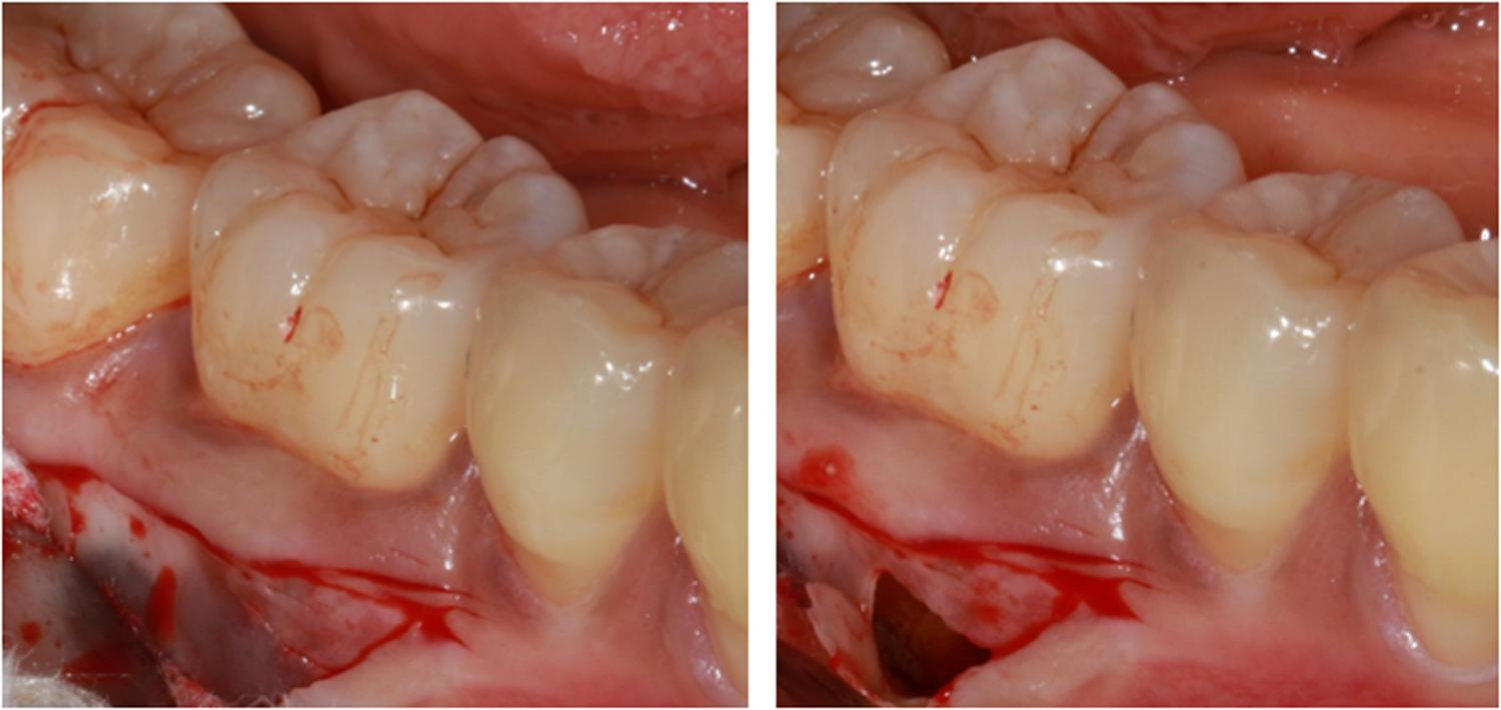
Case 1. Surgical exploration showing the bluish appearance of the cortical bone (A) and the underlying empty bone cavity (B).

The patient was followed over a 5‐year period, during which two additional surgical interventions were performed. The first involved the anterior region (#32) due to pain and tooth mobility, which subsided after the procedure. The second was indicated by recurrence of swelling near tooth #46. In both cases, surgical exploration again revealed empty bone cavities without epithelial lining. At the 5‐year follow‐up, the patient remains asymptomatic. Radiographically, the FCOD lesions appear stable, and the areas previously occupied by SBCs have shown progressive but incomplete ossification over time (Figure 3). Given clinical and radiological stabilization, no further surgical interventions are planned, and annual monitoring has been implemented.

**Figure 3:**
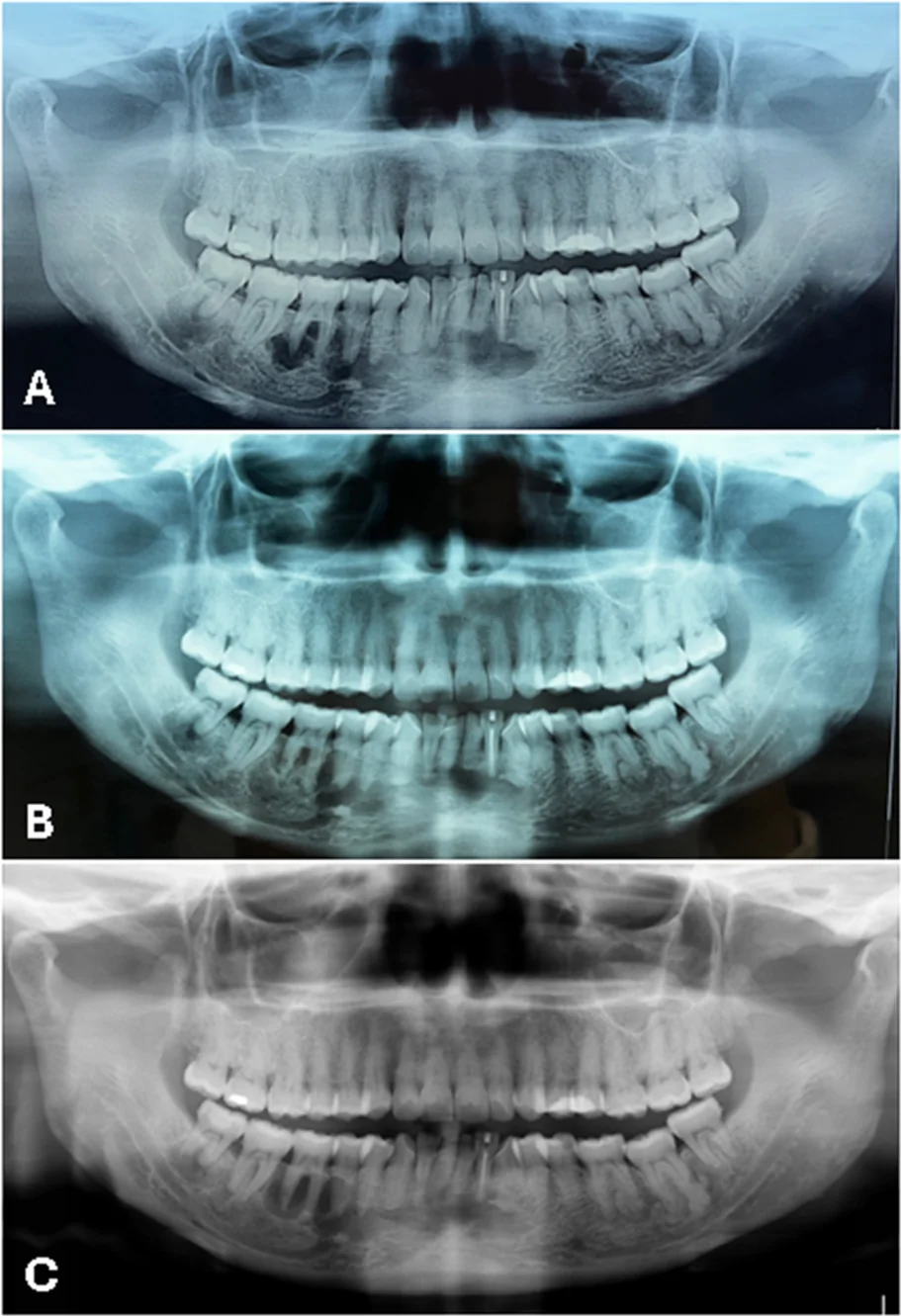
Case 1. Panoramic radiographs obtained 2 years (A), 4 years (B), and 5 years (C) after the first biopsy, showing partial bone healing of the largest lesion and spontaneous partial remineralization of the unilocular lesion in the #48 region.

### 2.2 Case 2

A 40‐year‐old woman of Congolese origin was referred to the Department of Oral Surgery following the incidental detection of a large, asymptomatic radiolucent lesion in the mandible. Clinical examination revealed no intraoral or extraoral swelling, and all teeth were responsive to vitality testing.

Panoramic radiography and CBCT revealed multiple mixed radiolucent–radiopaque lesions involving the apices of several mandibular and maxillary teeth, consistent with FCOD. In addition, a large multilocular radiolucency was observed in the left mandibular body (Figure 4), occupying the edentulous region of the missing second and third molars, extending mesially to the distal root of the first molar and posteriorly to the mandibular angle. The lesion showed irregular margins, thinning and focal perforation of the lingual cortical plate, mimicking the biological behavior of a more aggressive lesion. Scattered radiopaque foci were also noted within the lesion.

**Figure 4:**
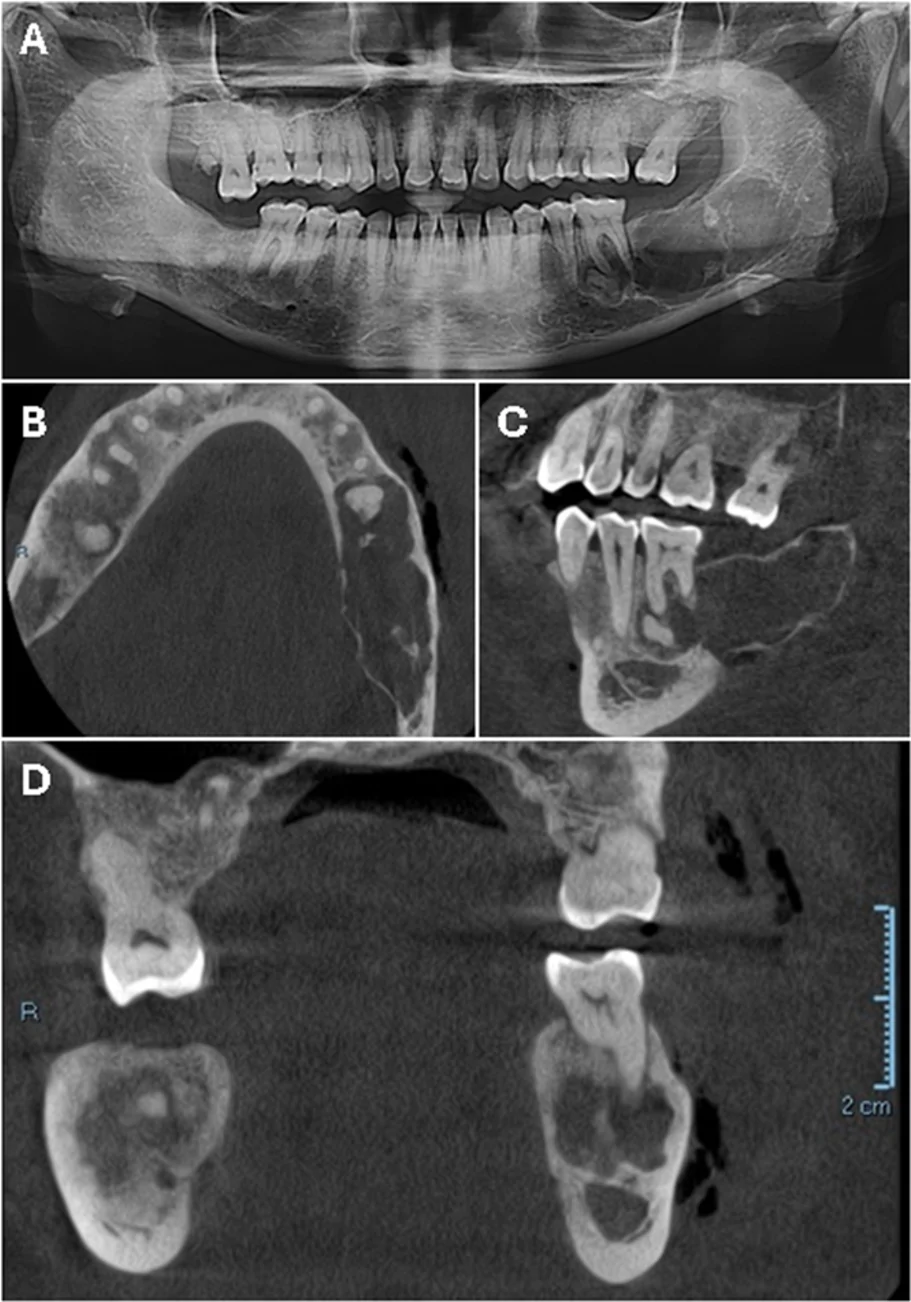
Case 2. Initial panoramic radiograph (A) and axial (B), sagittal (C), and coronal (D) CBCT views showing multiple mixed lesions around the apices of maxillary and mandibular teeth, as well as the radiolucent multilocular lesion in the left mandibular body.

Based on these findings, a provisional diagnosis of FCOD associated with a secondary SBC was established. Surgical exploration was performed to exclude a more aggressive pathology. Intraoperatively, an empty bone cavity without epithelial lining was observed (Figure 5), supporting the diagnosis of an SBC. A targeted biopsy was obtained from the posterior radiopaque area of the lesion, corresponding to the edentulous region of the missing third molar. Histopathological examination demonstrated a fibro‐osseous lesion consistent with FCOD (Figure 6). Taken together, the clinical, radiographic, surgical, and histopathological findings supported the diagnosis of FCOD associated with a secondary SBC. At the 6‐month follow‐up, panoramic imaging (Figure 7) demonstrated nearly complete osseous healing. Given the favorable evolution, annual radiographic monitoring has been instituted.

**Figure 5:**
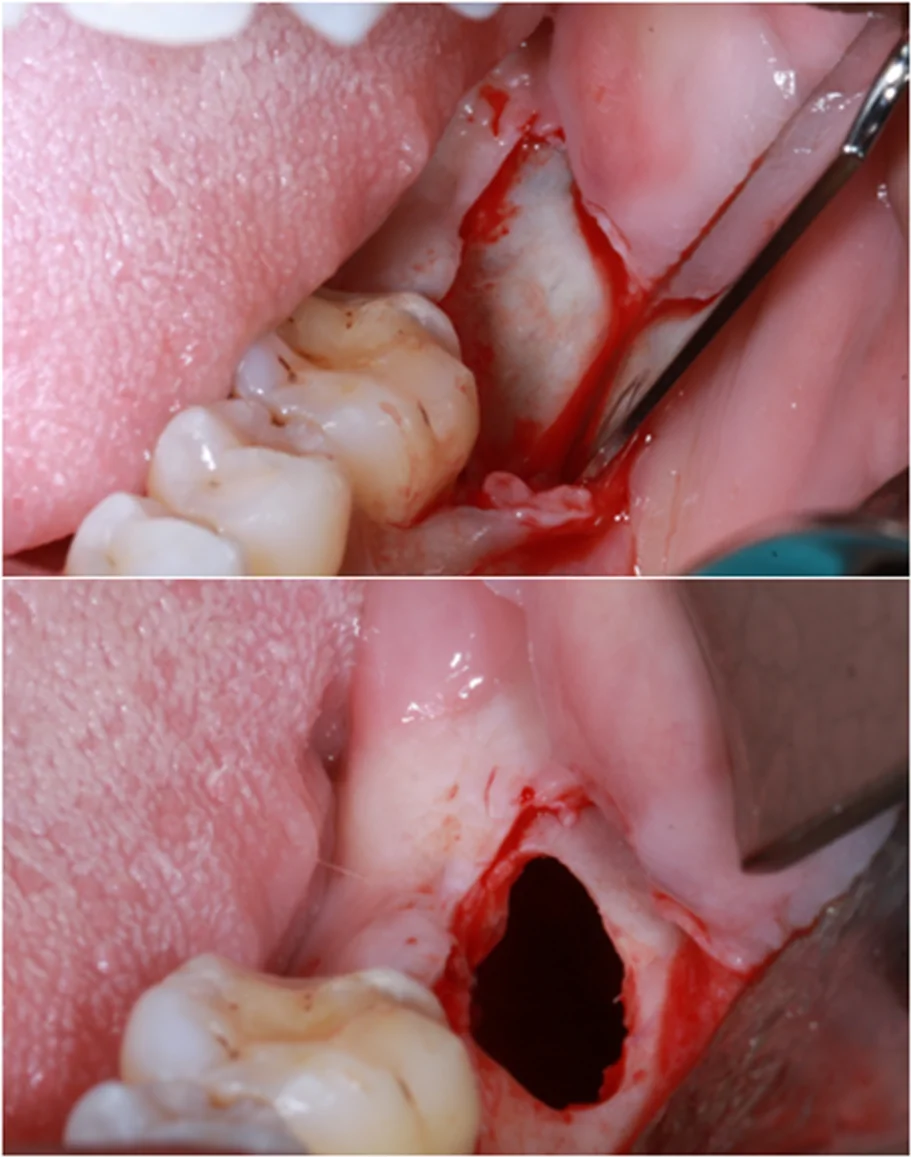
Case 2. Clinical appearance after mucoperiosteal flap elevation showing the bluish appearance of the cortical bone (A) and the underlying empty bone cavity without soft‐tissue lining (B).

**Figure 6:**
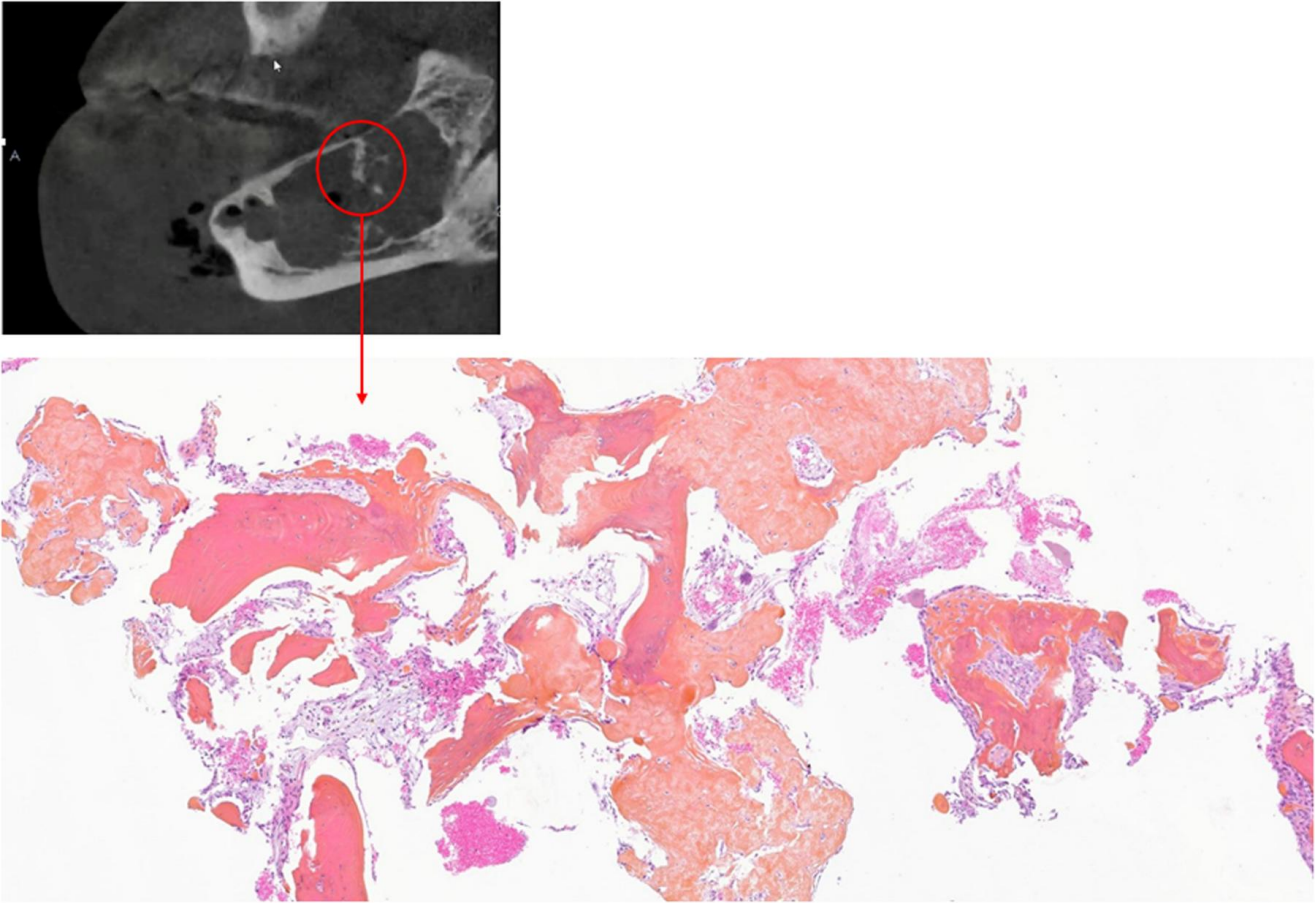
Case 2. On CBCT (A), a radiopaque area was identified within the radiolucent lesion in the left mandible; this portion was sampled for histological analysis. (B) Histological findings: a fibro‐osseous lesion composed of woven bone, associated with rare cementum‐like structures. The fibrous component consists of spindle‐shaped cells. In some areas, superficial deposits of immature bone were also observed (hematoxylin and eosin stain, original magnification).

**Figure 7:**
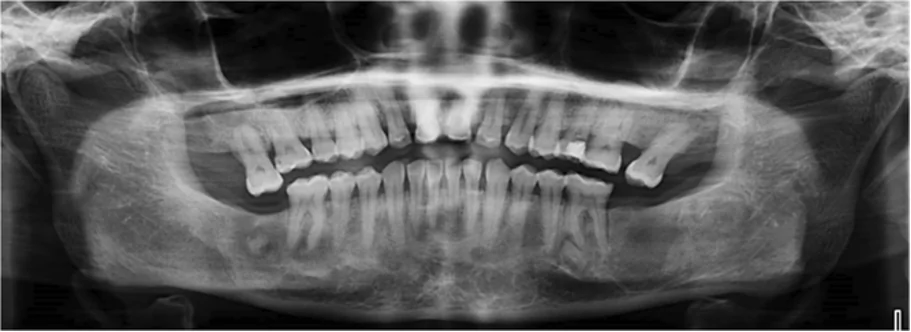
Case 2. Panoramic radiograph obtained 6 months postoperatively, showing that the radiolucent area corresponding to the secondary SBC had reached a bone density comparable to that of the surrounding healthy tissue, indicating progressive healing after surgical intervention.

## 3 Discussion

Fibro‐osseous lesions of the jaws are of particular relevance to oral and maxillofacial surgeons, given their key role in both diagnosis and management. Due to the wide spectrum of radiographic appearances, a thorough understanding of their characteristic features is essential for accurate diagnosis and appropriate treatment planning (Achararit et al. 2025). In most cases, the diagnosis of COD is established based on typical radiographic images, as patients are typically asymptomatic, and biopsy is generally unnecessary. However, COD may occasionally be complicated by secondary infections such as osteomyelitis (Kato et al. 2018; Graf et al. 2025) or by the development of SBCs. The association of COD and SBCs appears to fall within a broader group of non‐epithelial cystic changes arising in the context of benign fibro‐osseous lesions, including fibrous dysplasia, ossifying fibroma, and osteoblastoma (Hajjami et al. 2023; Zillo Martini et al. 2010). Melrose et al. were the first to report the co‐occurrence of COD and SBCs and to propose that these secondary SBCs may differ from their solitary counterparts. Since 1976, 69 patients with concomitant COD and SBCs have been reported in the literature. Including the two patients presented in this report, a total of 71 patients were included in our review (Table 1). This comprehensive review suggests that SBCs occurring in association with COD constitute a recognizable clinicoradiological manifestation within the COD spectrum, with features that consistently differ from those of solitary SBCs, rather than supporting the definition of a distinct clinicopathological entity. These observations are in agreement with the recent systematic review and meta‐analysis by Rave et al., which identified similar demographic, clinical, radiographic, and therapeutic characteristics among patients with FCOD‐associated SBCs (Rave et al. 2025). Their findings further support the concept that these lesions represent a recognizable manifestation within the spectrum of COD and emphasize the importance of distinguishing them from solitary SBCs.

**Table 1:** Publications reporting the association between SBC and COD in the literature, according to sex, age, ethnicity, clinical and radiological manifestations, management, and outcome. NS, not specified.

Study/author	Number of patients (*N*)/number of SBCs (*n*) gender mean age (years)	Patient ethnicity	Clinical manifestations	Radiological manifestations of SBCs	Outcome	Follow‐up duration
Melrose et al. (1976)	*N* = 14/*n* = 17 13 F 42 y	13 African 1 Oriental	Bone expansion (*n* = 4) Pain or a dull aching sensation (*n* = 5)	‐ Well‐defined ‐ Smaller SBC: Well delimited and unilocular ‐ Larger SBC: Multilocular	Complete healing (*n* = 6) Partial healing or enlargement (*n* = 3)	1–29 years (*n* = 9)
Higuchi et al. (1988)	*N* = 4/*n* = 4 1 F 41 y	NS	Asymptomatic (*n* = 2) Painless swelling (*n* = 2)	‐ Unilocular	NS	NS
Horner and Forman (1988)	*N* = 2/*n* = 2 1 F 37 y	Indian	Asymptomatic (*n* = 1) Painless swelling (*n* = 1)	‐ Well‐defined unilocular (*n* = 1) ‐ Multilocular (*n* = 1)	‐ Complete healing (*n* = 1) ‐ Healing (NS) (*n* = 1)	2 years 18 months
Miyauchi et al. (1995)	*N* = 1/*n* = 2 1 F 40 y	Japanese	Pain Swelling Bone expansion	‐ Multilocular with scalloped margins ‐ Ill‐defined area	‐ Partial healing (*n* = 1) ‐ Recurrence (*n* = 1)	14 months
Wakasa et al. (2002)	*N* = 1/*n* = 3 1 F 34 y	NS	Painful swelling (*n* = 1) Painless swelling (*n* = 1) Bone expansion	‐ Unilocular ‐ Bone expansion ‐ Thinning and focal cortical perforation	‐ Healing	4 years
Mahomed et al. (2005)	*N* = 7/*n* = 7 6 F 43 y	African	Pain (*n* = 4) Painful swelling (*n* = 1) Painless swelling (*n* = 1) Asymptomatic (*n* = 1)	‐ Multilocular (*n* = 3) ‐ Scalloped margins ‐ Unilocular (*n* = 4)	NS	Lost to follow‐up (*n* = 5)/no information (*n* = 2)
Mupparapu et al. (2005)	*N* = 1/*n* = 1 1 F 41 y	African	Asymptomatic	‐ Multilocular	NS	3 months
Zillo Martini et al. (2010)	*N* = 3/*n* = 3 F 40 y	African American	Asymptomatic (*n* = 1) Painless swelling (*n* = 2)	‐ Unilocular (*n* = 2): Vestibular and lingual bone expansion (*n* = 1) ‐ Multilocular (*n* = 1): Vestibular bone expansion (*n* = 1)	Partial healing (*n* = 1) Recurrence, then partial healing after surgery (*n* = 1)	5 years (*n* = 1) 18 months (*n* = 1) Lost to follow‐up (*n* = 1)
Chadwick et al. (2011b)	*N* = 23/*n* = 23 20 F 45 y	Not specified	NS	‐ Cortical thinning (*n* = 18; 78.2%) ‐ Bone expansion (*n* = 14; 60.8%) ‐ Scalloped margins (*n* = 19; 82.6%)	NS	NS
Rao et al. (2011)	*N* = 1/*n* = 1 F 41 y	Asian	Painful swelling	‐ Multilocular with scalloped margins ‐ Ill‐defined area ‐ Vestibular and lingual bone expansion	Asymptomatic No radiographic data	6 months
Peacock et al. (2015)	*N* = 6/*n* = 6 6 F NS	African American	Painful swelling (*n* = 5) Asymptomatic (*n* = 1)	No specific data regarding secondary SBC with COD	1 recurrence	1–8 years
Kojima et al. (2020)	*N* = 1/*n* = 2 F 39 y	Japanese	Asymptomatic	‐ Multilocular with scalloped margins (*n* = 1) ‐ Multilocular with irregular margins (*n* = 1) ‐ Thinning and focal cortical perforation	Enlargement with bone expansion (*n* = 2)	27 months
Lee et al. (2020)	*N* = 1/*n* = 2 F 48 y	Chinese	Painful swelling (*n* = 1) Asymptomatic (*n* = 1)	‐ Unilocular (*n* = 1) ‐ Multilocular (*n* = 1) ‐ Bone expansion, scalloped margins	NS	NS
Hajjami et al. (2023)	*N* = 1/*n* = 2 F 31 y	African	Painless swelling (*n* = 1) Asymptomatic (*n* = 1)	‐ Multilocular, ill‐defined area ‐ Bone expansion	Enlargement, new occurrence	11 years
Berberi (2024)	*N* = 1/*n* = 1 F 46 y	Oriental	Painless swelling (*n* = 1)	‐ Unilocular ‐ Bone expansion ‐ Thinning and focal cortical perforation	NS	NS
Rollin and Taihi (2024)	*N* = 2/*n* = 2 2 F 41 y	African	Asymptomatic (*n* = 1) Painful swelling (*n* = 1)	‐ Unilocular (*n* = 2) ‐ Bone expansion (*n* = 2) ‐ Thinning of cortical bone (*n* = 2)	Partial healing (*n* = 1) Complete healing (*n* = 1)	9 months 2 years
Present report (2026)	*N* = 2/*n* = 3 2 F 42 y	Martinican African	Painful swelling (*n* = 1) Asymptomatic (*n* = 1) Pain and tooth mobility (*n* = 1)	Multilocular (*n* = 2) with thinning and focal cortical perforation Unilocular (*n* = 1)	Partial healing (*n* = 2) Complete healing (*n* = 1)	4 years 6 months

First, several mechanisms have been proposed to explain the development of secondary SBCs in COD‐affected bone. One hypothesis suggests that rapid bone turnover and disorganized fibro‐vascular proliferative tissue in COD could lead to venous stasis and interstitial fluid accumulation (Rao et al. 2011; Lee et al. 2020; Miyauchi et al. 1995), ultimately forming a bone cavity. Alternatively, some authors consider these SBCs to be secondary degenerative changes within pre‐existing COD, suggesting a post‐dysplastic origin rather than a distinct lesion (Miyauchi et al. 1995). As such, and in line with the 2022 WHO classification (Vered and Wright 2022), these should be referred to as secondary SBCs when associated with COD, to distinguish them from true solitary SBCs. This could explain the tendency of these lesions to become symptomatic and to display radiological features of aggressiveness, such as irregularity and cortical perforation. It may also account for cases of recurrence following surgical treatment and the reappearance of these lesions.

Several consistent differences distinguish solitary SBCs from SBCs associated with COD (Table 2). Compared with solitary SBCs, which typically occur in young patients with no sex predilection, secondary SBCs are reported predominantly in middle‐aged women, reflecting the epidemiology of COD. They are also more frequently multiple and preferentially involve the posterior mandible, although anterior mandibular and rare maxillary locations have been described (Melrose et al. 1976), including one of our cases. In contrast, solitary SBCs occur mostly in the anterior mandible (Choi et al. 2022). Clinically, solitary SBCs are usually asymptomatic incidental findings, whereas COD‐associated SBCs more often become symptomatic, presenting with pain, swelling, or persistent discomfort. This observation is supported by our literature review, encompassing all documented cases of concomitant COD and SBCs, in which only 10 patients were asymptomatic. Radiographically, solitary SBCs typically present as well‐defined, unilocular, and homogeneous radiolucent lesions, often exhibiting scalloping between adjacent tooth roots (Vered and Wright 2022). Circumscribed secondary SBCs could share the same radiological appearance, whereas larger lesions are often multilocular and are more likely to cause cortical thinning or perforation, bone expansion and demonstrated a loss of lamina dura more often than solitary SBCs (Chadwick et al. 2011a). Secondary SBCs also often demonstrate mixed radiolucent–radiopaque appearances reflecting the underlying fibro‐osseous lesion. Together, these features explain why these lesions frequently mimic more aggressive jaw pathology and justify surgical exploration for definitive diagnosis. These surgical explorations consistently reveal empty bone cavities, consistent with SBCs. In all reviewed cases, a surgical approach was undertaken, involving either curettage of the cavity walls (reported in 2 studies) or surgical exploration with biopsy of the cavity lining or bone window (reported in 15 studies). This is consistent with solitary SBC treatment, which involves a surgical approach with or without curettage of the walls to promote revascularization of the lesion (Pérez‐Iglesias et al. 2021).

**Table 2:** Comparison between solitary simple bone cysts (solitary SBCs) and secondary simple bone cysts associated with cemento‐osseous dysplasia (secondary SBCs).

	Primary SBC	Secondary SBC associated with COD
Epidemiology	Adolescents and young adults	Middle‐aged adults, often female
Location	Often anterior mandible	Often posterior mandible, areas affected by COD
Radiological appearance	Well‐defined, homogeneous radiolucency Unilocular	Radiolucency less homogeneous, sometimes signs of aggressiveness Unilocular or multilocular
Symptoms	Asymptomatic	May become symptomatic (pain, tooth mobility, and asymmetry)
Treatment	Single surgical exploration and curettage	One or more surgical explorations with curettage.
Macroscopic appearance	Empty bone cavity	Empty bone cavity
Clinical course and recurrence rate	Rapid and complete healing after curettage. No recurrence	Partial or complete healing. Higher risk of recurrence

Healing patterns also appear to differ from those observed in solitary SBCs (Mupparapu et al. 2005). Whereas isolated SBCs generally heal predictably after surgical exploration, COD‐associated SBCs often demonstrate delayed, incomplete, or recurrent healing. In our review, most reported cases showed partial (*n* = 9) or complete radiographic improvement (*n* = 9) following surgery, although recurrence or the development of new SBCs was documented in several patients (Hajjami et al. 2023; Mahomed et al. 2005). Our first case illustrates this challenge, as incomplete exploration of a multilocular lesion may have contributed to recurrence. Similar observations have been reported in multilocular SBCs of the long bones, where complete exploration and removal of intralesional septa reduced recurrence rates (Aiba et al. 2018). Although direct evidence remains limited in the jaws, these findings suggest that meticulous exploration of all loculations, associated with septal resection, may improve outcomes in extensive multilocular lesions associated with COD.

Because symptomatic improvement has been inconsistently reported (Zillo Martini et al. 2010; Rollin and Taihi 2024), and radiographic healing may be prolonged, regular clinical and radiographic follow‐up is recommended. After initial postoperative monitoring, annual surveillance appears appropriate to detect recurrence, the development of new cystic lesions, or progression of pre‐existing lesions. Similarly, the recent meta‐analysis regarding FCOD‐associated SBCs reported complete radiographic healing in 52% of cases and partial healing in 32% after surgical exploration, while recurrence occurred in approximately 16% of followed patients after a mean follow‐up of 6.6 years, highlighting the need for long‐term surveillance (Rave et al. 2025).

Finally, among the 71 patients included in our review, 39 (55%) of secondary SBCs occurred in patients with FCOD, suggesting that the florid form provides the most favorable environment for secondary SBC formation (Melrose et al. 1976; Peacock et al. 2015). Thus, SBCs appear to be much more frequently linked to the florid form of COD. This is likely related to the fact that FCOD induces extensive bone remodeling, characterized by poor vascularization and impaired drainage, which promote bone cavitation. Its multifocal distribution and advanced evolutionary stage make it a far more favorable setting than the focal or periapical forms, although the exact pathophysiological mechanisms remain to be clarified.

## 4 Conclusion

The association of COD and SBCs, although uncommon, represents a recognizable clinicoradiological manifestation within the spectrum of COD rather than a distinct clinicopathological entity. Compared with solitary SBCs, these lesions show consistent differences in patient profile, distribution, clinical behavior, radiographic features, and healing pattern. Recognition of this association is important because it may mimic more aggressive jaw lesions and frequently justifies surgical exploration to establish the diagnosis and promote healing. Our findings, consistent with the literature, support the classification of these cysts as secondary SBCs within the COD spectrum. Further studies are needed to elucidate the underlying pathogenesis and long‐term outcomes of these combined lesions.

## Author Contributions

**Victor Voisin:** conceptualization, investigation, writing – original draft, writing – review and editing. **Paul Galvez:** writing – review and editing. **Jean‐Christophe Fricain:** supervision, writing – review and editing. **Mathilde Fénelon:** conceptualization, resources, supervision, writing – review and editing.

## Funding

The authors have nothing to report.

## Consent

Written informed consent was obtained from both patients for the publication of these case reports, including intraoral clinical and radiographic images. The patients are not identifiable, and all data have been fully anonymized in accordance with the ethical standards of Clinical and Experimental Dental Research and the Declaration of Helsinki. Ethical committee approval was not required for these two anonymized case reports.

## Conflicts of Interest

The authors declare no conflicts of interest.

## Declaration of Generative AI and AI‐Assisted Technologies in the Writing Process

During the preparation of this work, the authors used ChatGPT, an AI‐powered language tool, to refine grammar, correct spelling errors, and enhance sentence structure for improved clarity and readability. After utilizing this tool, the authors carefully reviewed and edited the content to ensure its accuracy and integrity. The authors take full responsibility for the scientific content and originality of this publication.

## Data Availability

The data that support the findings of this study are available on request from the corresponding author. The data are not publicly available due to privacy or ethical restrictions.
